# Single-molecule localization microscopy as a tool to quantify di/oligomerization of receptor tyrosine kinases and G protein-coupled receptors

**DOI:** 10.1016/j.molpha.2025.100033

**Published:** 2025-03-26

**Authors:** Katie L. Sharrocks, Aisha M. Swaih, Aylin C. Hanyaloglu

**Affiliations:** 1 The Francis Crick Institute, London, UK; 2 AstraZeneca, Cambridge, UK; 3 Department of Metabolism, Digestion and Reproduction, Imperial College London, London, UK

**Keywords:** Dimerization, G protein-coupled receptors, Oligomerization, Receptor tyrosine kinases, Single-molecule localization microscopy, Super-resolution microscopy

## Abstract

Dimerization and oligomerization of membrane receptors, including G protein-coupled receptors and receptor tyrosine kinases, are fundamental for regulating cell signaling and diversifying downstream responses to mediate a range of physiological processes. Receptor di/oligomers play roles in diverse facets of receptor function. Changes in receptor di/oligomers have been implicated in a range of diseases; therefore, better understanding of the specific composition and interactions between receptors in complexes is essential, especially for the development of di/oligomer-specific drugs. Previously, different optical microscopy approaches and proximity-based biophysical assays have been used to demonstrate di/oligomerization of membrane receptors. However, in recent years, single-molecule super-resolution microscopy techniques have allowed researchers to quantify and uncover the precise dynamics and stoichiometry of specific receptor complexes. This allows the organization of membrane protein receptors to be mapped across the plasma membrane to explore the effects of factors such as ligands, effectors, membrane environment, and therapeutic agents. Quantification of receptor complexes is required to better understand the intricate balance of distinct receptor complexes in cells. In this brief review, we provide an overview of single-molecule approaches for the quantification of receptor di/oligomerization. We will discuss the techniques commonly employed to study membrane receptor di/oligomerization and their relative advantages and limitations.

**Significance Statement:**

Receptor di/oligomerization plays an important role in their function. For some receptors, di/oligomerization is essential for functional signaling, whereas for others, it acts as a mechanism to achieve signaling pleiotropy. Aberrant receptor di/oligomerization has been implicated in a wide range of diseases. Single-molecule super-resolution microscopy techniques provide convincing methods to precisely quantify receptor complexes at the plasma membrane. Understanding receptor complex organization in disease models can also influence the targeting of specific monomeric or oligomeric complexes in therapeutic strategies.

## 1 Introduction

### 1.1 Membrane protein receptors

Membrane proteins such as receptor tyrosine kinases (RTKs) and G protein-coupled receptors (GPCRs) are fundamental components of signal transduction pathways that regulate a wide range of physiological processes. The superfamily of GPCRs, with more than 800 members, are grouped based on sequence homology into class A (rhodopsin), B (secretin and adhesion), C (glutamate), and F (frizzled), with each class exhibiting distinct structural or functional characteristics (Fredriksson et al, 2003; Yang et al, 2021). Despite their differences, all GPCRs share a common architecture of 7 transmembrane helices, and upon receptor activation, conformational changes occur, which allow G protein activation and recruitment of *β*-arrestin adapter proteins (Oldham and Hamm, 2008; Hauser et al, 2021). GPCR signaling is diversified by a range of mechanisms, including G protein type, signaling bias, compartmentalization of receptor signaling, and di/oligomerization.

RTKs are single-pass transmembrane receptors with an extracellular ligand binding domain and are characterized by their cytoplasmic tyrosine kinase domain. When phosphorylated by the tyrosine kinase domain, the C-terminal tail acts as a site for the recruitment of proteins, leading to stimulation of a range of signaling pathways including the mitogen-activated protein kinase pathway. There are 58 human RTKs, which are grouped into 20 subfamilies and are key regulators in metabolism, cell proliferation, and survival (Robinson et al, 2000; Hubbard and Miller, 2007; Lemmon and Schlessinger, 2010). Both GPCRs and RTKs are successful and highly tractable drug targets. Although RTKs represent a key therapeutic target in cancer, over 34% of the Food and Drug Administration drugs target GPCRs, treating a wide range of diseases from diabetes and obesity to neurological disorders (Hauser et al, 2017; Du and Lovly, 2018; Yang et al, 2021). Therefore, it is essential that the structural and functional characteristics of these families of receptors are understood to facilitate new therapeutic ways to target these receptors.

### 1.2 Receptor di/oligomerization

**Fig. 1:**
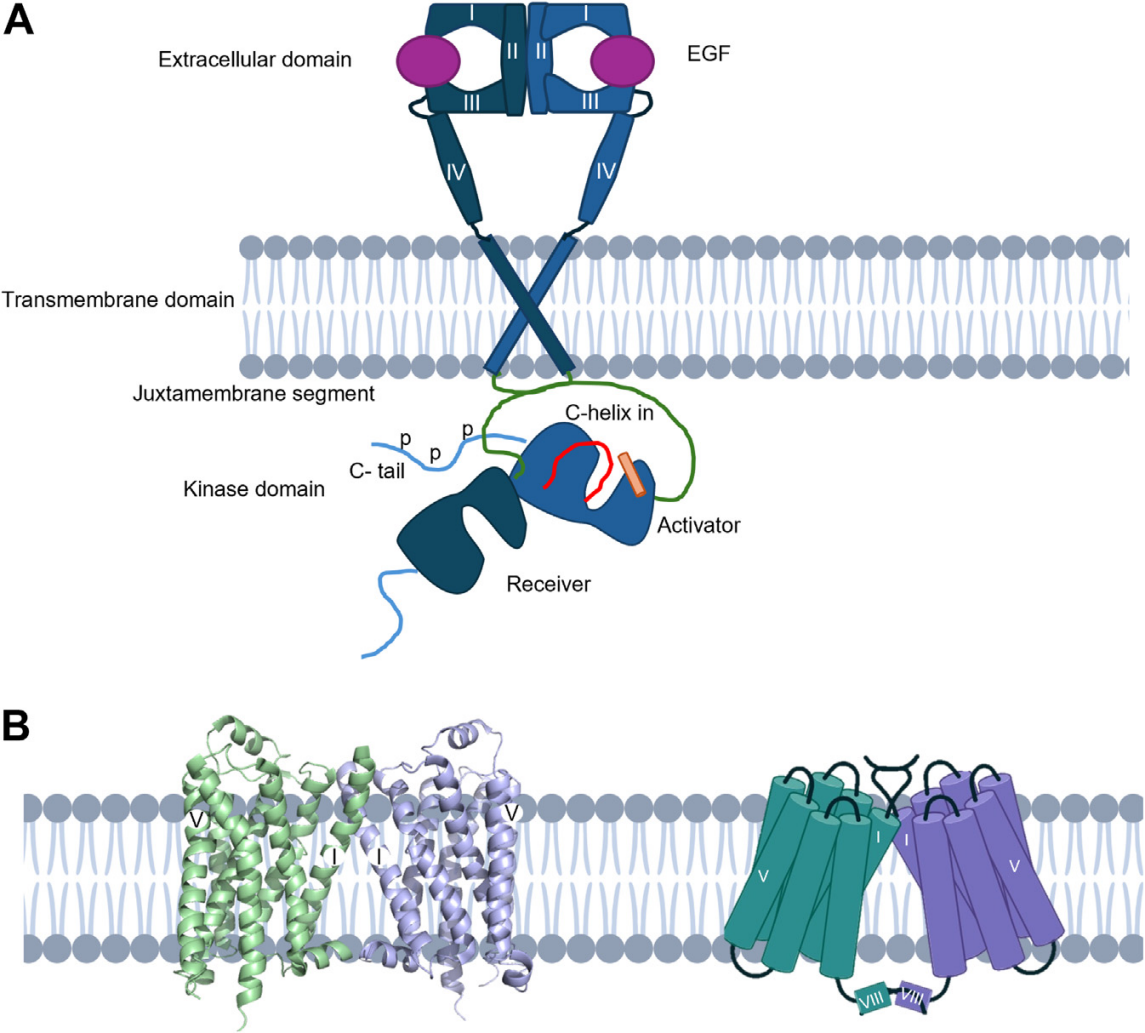
Example of RTK and GPCR dimers. (a) Epidermal growth factor receptor (EGFR) dimerization upon activation with EGF. The EGF ligand does not play a direct role in the dimer interface but stabilizes the active conformation that enables asymmetric dimerization of the kinase domain and phosphorylation of the C-tail. (b) G protein-coupled receptor dimer interfaces vary greatly and here an example of a class A GPCR is shown; the *β*1-adrenergic receptor (PDB: 4GPO; Huang et al, 2013). Depicted as the ribbon structure and as a schematic. The dimer interface involves transmembrane helices I, II, VIII and extracellular loop 1 (ECL1).

In general, RTKs are required to form dimers for signal activation. Some RTKs can form oligomers in the absence of activating ligands. However, for most RTKs, ligand-induced receptor dimerization causes conformational changes in the extracellular domain, resulting in the stabilization of specific active di/oligomeric receptor complexes, eventually facilitating the activation of intracellular kinase domains (Fig. 1a). Ligands play varying roles in RTK dimerization, ranging from acting effectively as a crosslink between receptors in a dimer, to playing no direct role in the dimerization interface, but instead inducing changes or stabilizing conformations (Schlessinger, 2000; Lemmon and Schlessinger, 2010). For example, in the activation of ErbB receptors (epidermal growth factor receptor [EGFR], ErbB2/HER2, ErbB3/HER3, and ErbB4/HER4), ligands play no direct role in the dimerization interface, instead causing conformational changes in the extracellular domain to allow receptor dimerization. In EGFR activation, for example, dimerization of the extracellular domains following ligand binding results in asymmetric dimerization and activation of the intracellular tyrosine kinases domains and autophosphorylation, allowing the activation of downstream signaling proteins (Schlessinger, 2000; Zhang et al, 2006; Red Brewer et al, 2009; Lemmon and Schlessinger, 2010). Although much of the focus has been on understanding EGFR activation mechanisms through kinase domain dimerization, receptor oligomers composed of multiple receptors also likely play a key role in EGFR signaling (Clayton et al, 2005; Zhang et al, 2006; Mudumbi et al, 2024).

In contrast, many GPCRs are thought to not require dimerization for signal activation and can function effectively as monomeric units (Whorton et al, 2007). Class C GPCRs are obligate dimers; however, many types of other GPCRs have been shown to form homodimers or heterodimers with other GPCRs, as well as forming higher-order oligomeric complexes (Angers et al, 2000; Pin et al, 2007; Ferré et al, 2009, 2014; Gomes et al, 2016; Asher et al, 2021). Historically, the existence of the dimers of the large class A family of GPCRs was highly debated but has now been widely reported to form di/oligomers. Much of the controversy arose from the limitations of the methods used to study these dimers and the inability to determine the functional physiological relevance of class A GPCR di/oligomerization in vivo (Asher et al, 2017; Felce et al, 2018; Sierra et al, 2020). However, in recent years, numerous studies have demonstrated the physiological relevance and/or the role of GPCR dimers in disease (Rivero-Müller et al, 2010; Gomes et al, 2016; Moreno-Delgado et al, 2020). The assessment of the dynamics of several class A receptor dimers in different heterologous systems has revealed that it is likely that a dynamic equilibrium forms between monomers and di/oligomers, meaning these complexes are often short-lived. However, the stability of the di/oligomeric complex is likely receptor and context-dependent (Hern et al, 2010; Kasai et al, 2011, 2018; Calebiro et al, 2013; Kasai and Kusumi 2014; Tabor et al, 2016). The most important role of GPCR di/oligomerization appears to be the profound impact that modulating the relative levels of different receptor complexes can have on signal activity via several mechanisms. Despite the transient nature of di/oligomerization of some GPCRs, it can dramatically impact the function of receptors, including by altering ligand efficacy or affinity, G protein coupling selectivity, and G protein or *β*-arrestin activation (Ferré et al, 1991; George et al, 2000; Rodrí;guez-Frade et al, 2001; Ferré et al, 2009; Ge et al, 2017; Liu et al, 2022; Sharrocks et al, 2024). The class C metabotropic glutamate receptors highlight the profound impact that homo/heterodimerization can have on receptor pharmacology and the importance of studying GPCR dimerization, particularly for therapeutic targeting of specific di/oligomer complexes (Du et al, 2021; Wang et al, 2023; Lin et al, 2024). Additionally, a range of functions beyond signaling diversification have been proposed for GPCR di/oligomerization. Dimerization of GPCRs may be involved in the folding, maturation, and trafficking of GPCRs to the plasma membrane (Bulenger et al, 2005).

The nature of interactions and interfaces between receptor di/oligomers varies greatly between different GPCRs (Fig. 1b). Some GPCR dimers have very small interfaces consisting of only a few residue contacts, such as the apelin receptor, whereas others, such as the Ste2 dimer, form receptor interfaces with extensive contacts, spanning large areas of transmembrane domains (Velazhahan et al, 2022; Yue et al, 2022). However, the extracellular loops in many types of GPCRs and the large extracellular domains in class C GPCRs appear to play key roles in many GPCR-dimer interfaces (Huang et al, 2013; Papasergi-Scott et al, 2020; Velazhahan et al, 2022).

Receptor interfaces of dimers are not rigid but are instead flexible and dynamic (Kasai and Kusumi, 2014; Diwanji et al, 2021). Additionally, several different di/oligomer conformations have been demonstrated, even for the same receptor complex but under different conditions or with different receptor mutations for both ErbB receptors and GPCRs (Jonas et al, 2015; van Lengerich et al, 2017; Dijkman et al, 2018; Xue et al, 2019). For example, ErbB receptor-dimer interfaces, oligomer organization, and heterocomplex type have been shown to be influenced by stimulation with different ligands (van Lengerich et al, 2017; Huang et al, 2021).

A further mechanism to connect and diversify signal transduction pathways is through GPCR-RTK crosstalk via either indirect interactions or through the formation of GPCR-RTK complexes, such as the serotonin 5-hydroxytryptamine and fibroblast growth factor receptor 1 heteroreceptor complexes (Borroto-Escuela et al, 2016). This highlights the complexity and the importance of understanding the detailed molecular makeup of distinct receptor complexes.

### 1.3 Role of di/oligomerization in physiology and disease

The physiological relevance of signaling changes as a result of GPCR di/oligomerization is less well defined. However, a range of GPCR di/oligomers have been shown to play a key role in modulating a range of processes. For example, the angiotensin II type I and the prostaglandin F2*α* heteromers have been suggested to be important in the regulation of blood pressure (Goupil et al, 2015). Additionally, the formation of homo/heteromers of the gonadotrophin receptors—luteinizing hormone receptor and follicle-stimulating hormone receptor—is important for reproductive health and fertility (Agwuegbo and Jonas, 2018).

Many types of GPCR di/oligomers have been implicated in a range of diseases. Some of the most prominent examples of GPCR di/oligomers involved in disease are in the central nervous system. This includes D2 dopamine receptor homodimers and heteromers, with the A2A adenosine receptor implicated in neurological diseases such as Parkinson disease and schizophrenia, and serotonin (5-hydroxytryptamine) 1A and orexin 1 receptor heterodimers involved in depression pathogenesis (Wang et al, 2010; Bonaventura et al, 2015; Fuxe et al, 2015; Borroto-Escuela et al, 2018; Zhang et al, 2022).

In the case of RTKs, receptor dimerization is usually tightly regulated, and constitutive activation of RTKs can lead to oncogenesis (Schlessinger, 2000; Lemmon and Schlessinger, 2010). ErbB family of receptors is often dysregulated in cancer. Mutations that result in changes to the kinase domain or the extracellular domain can result in altered dimerization and activation, including ligand-independent dimerization, leading to hyperactivation and aberrant signaling in cancer (McDonell et al, 2015; Du and Lovly 2018). Additionally, it is possible that the rewiring of signaling through altering EGFR dimerization mechanisms and forming specific receptor complexes may be a way for lung cancers to develop therapeutic resistance (Gupta et al, 2024).

Given the important role of receptor di/oligomerization in modulating receptor function and disease, understanding the specific composition of receptor complexes is fundamental. This information could potentially facilitate the design of complex specific drugs. Historically, a range of techniques, including biochemical, biophysical, computational, and high-resolution structural approaches, have been used to study receptor di/oligomerization (Cochet et al, 1988; Hebert et al, 1996; Salom et al, 2006; Guo et al, 2008; Selent and Kaczor 2011; Cottet et al, 2012). This review will explore the advances in spectroscopy and microscopy approaches, with a focus on single-molecule localization super-resolution microscopy, evaluating their advantages as tools to precisely map and track receptor complexes at the plasma membrane.

## 2 Microscopy and spectroscopy approaches to study receptor di/oligomerization

### 2.1 Resonance energy transfer approaches

Proximity-based resonance energy transfer (RET) live cell assays are widely used to study protein-protein interactions. The RET efficiency is dependent upon the distance between the donor and the acceptor, allowing an estimation of proximity between the 2 proteins. Historically, tagged receptors have been extensively used in fluorescence and bioluminescence resonance energy transfer (FRET and BRET) assays to study receptor homodimerization and heterodimerization, as well as GPCR-RTK heteroreceptor complexes (Cottet et al, 2012; Borroto-Escuela et al, 2013; Martínez-Pinilla et al, 2015).

RET techniques have been incorporated into microscopy approaches to assess the structure and dynamics of receptor interactions. Single-molecule FRET (smFRET) is a method that tracks the FRET efficiency of single donor and acceptor molecules, with the distance between the molecules inversely proportional to the FRET efficiency. This allows the tracking of receptor interactions and multiple conformations of receptor complexes at the plasma membrane and has been used to demonstrate dimerization of a wide range of receptors in live cells (Asher et al, 2021). As with all FRET- and BRET-based approaches, energy transfer only occurs when tagged receptors are within 10 nm of each other, which may be on the scale of some tagged receptors themselves. Additional considerations such as the location of the tags on the receptor may also influence RET efficiency (Pfleger and Eidne, 2006).

Fluorescence lifetime imaging microscopy (FLIM) can be combined with FRET to allow for the measurement of protein proximity within <10 nm with high spatial resolution and sensitivity. This technique monitors the time donor fluorophores stay in the excited state before emitting photons and returning to the ground state, with FRET between donor and acceptor reducing this lifetime. This approach has been used to quantify the heterodimerization of ErbB receptors HER2 and HER3 in breast cancer patients and may be a biomarker for metastatic relapse, which is independent of HER2 expression, with a clinical trial assessing this currently ongoing (Barber et al, 2013; Weitsman et al, 2016). This method has also been used to assess EGFR dimerization and oligomerization in inactive and active forms and uncover the conformation of EGFRs following activation (Clayton et al, 2005; Webb et al, 2008).

A related technique is fluorophore localization imaging with photobleaching. It can achieve ∼6 nm resolution by exploiting photobleaching to determine the separation between molecules by the localization of single molecules through monitoring emitting fluorophores during photobleaching using total internal reflection fluorescence (TIRF) microscopy (Needham et al, 2013, 2015, 2016). This technique has been used to assess EGFR dimerization and oligomerization, including constructing models of the structure and mechanisms of assembly of EGFR oligomers with distinct conformations, such as ligand-free or ligand-bound EGFR oligomers (Needham et al, 2016; Iyer et al, 2024). Both FLIM-FRET and fluorophore localization imaging with photobleaching offer increased sensitivity compared with traditional FRET to allow high-resolution interactions between individual molecules to be monitored. This level of resolution allows distinct conformations of receptor oligomers such as EGFR to be modeled by distinguishing between complexes that have different separation distances between fluorophores bound to receptors (Iyer et al, 2024).

Beyond RET approaches, developments in spectroscopy and microscopy techniques in recent years have expanded the detail that can be obtained about receptor complexes through both high spatial and temporal resolution. In practice, many of these approaches are combined, such as the use of single-particle tracking in combination with FLIM-FRET, to allow for the accurate quantification of receptor complexes as well as monitoring receptor dynamics.

### 2.2 Single-particle tracking

Single-particle tracking (SPT) uses TIRF microscopy and fluorophores such as SNAP-tags to allow direct visualization of cell surface receptors and their diffusion across the membrane. The trajectories and intensities of individual particles are tracked in a time-resolved manner. This approach has the advantage of being carried out on live cells, allowing receptor dynamics to be visualized. However, it requires very low receptor expression levels or labeling within the area being imaged to allow individual receptors to be resolved. Although this is cell type- and receptor-specific, this may be below physiologically relevant levels in certain tissues. Despite this, dimerization of several different GPCRs has been precisely monitored to achieve high spatiotemporal resolution (Hern et al, 2010; Kasai et al, 2011, 2018; Calebiro et al, 2013; Kasai and Kusumi 2014; Tabor et al, 2016; Möller et al, 2020). SPT and FRET have been used to study RTK dimerization including monitoring receptor dynamics after ligand activation and how EGFR variants impact this (Teramura et al, 2006; Chung et al, 2010; Valley et al, 2015). Additionally, single-molecule imaging approaches may have useful drug discovery applications. A SPT-based approach has been recently developed to screen a large panel of drugs that target transmembrane protein receptors, such as GPCRs and RTKs, including identifying compounds that affect receptor clustering or internalization (Watanabe et al, 2024). This highlights the importance of using single-molecule methods in studies furthering understanding of clinical drug mechanisms of action. Additionally, combining SPT with techniques such as FLIM-FRET and smFRET can be a powerful way to verify interactions while providing high temporal resolution. This combination of smFRET allows high-resolution SPT methods that do not rely on diffraction-limited colocalization approaches to assess receptor dimerization (Asher et al, 2021). SPT has been especially useful for achieving high temporal resolution for individual receptors, providing a higher level of detail beyond bulk BRET or FRET approaches that give an average of all interactions in the sample and may miss the dynamics of individual molecules.

### 2.3 Fluorescence correlation spectroscopy

Fluorescence correlation spectroscopy (FCS) is based on measuring the fluctuation of a diffraction-limited fluorescent spot to calculate a diffusion coefficient. FCS is often combined with photon counting histograms, which can estimate molecular brightness, and can then be used to correlate the fluorescence of a complex to a specific oligomer composition. This has been used to demonstrate the dimerization of several different GPCRs and RTKs and their dynamics and diffusion in the plasma membrane monitored (Briddon et al, 2004, 2008, 2018; Liu et al, 2007; Saffarian et al, 2007; Herrick-Davis et al, 2013). This approach requires low receptor expression levels and carefully selected controls to accurately determine oligomerization and it may be challenging to capture higher-order oligomers and accurately quantify a specific oligomer composition. However, it has the advantage of being able to assess receptor dynamics and can be carried out in live cells. It is possible to combine FCS with super-resolution microscopy to achieve both high spatial and temporal resolutions (Sankaran and Wohland, 2023). Additionally, the use of fluorescence cross-correlation spectroscopy, which assesses interactions between 2 fluorescent spots within a detection volume, in combination with pulsed-interleaved excitation, can further improve the specificity of FCS to assess receptor di/oligomerization with higher temporal resolution (Endres et al, 2013; Smith 2015).

## 3 Introduction to single-molecule localization microscopy

**Fig. 2:**
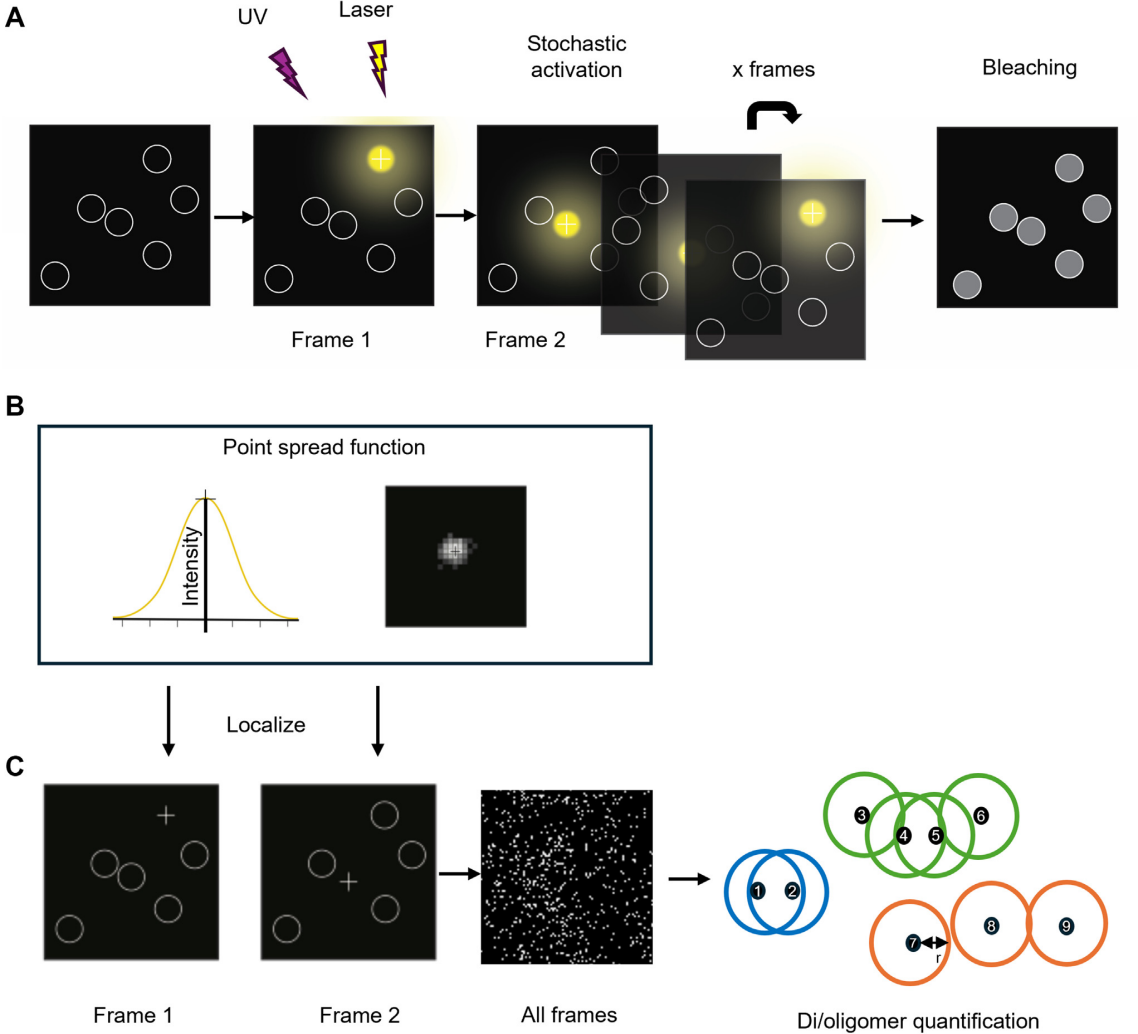
Principles of single-molecule localization microscopy. (a) Temporal separation of emissions from individual fluorophores allows the separation of point spread functions (PSF) of distinct fluorophores to achieve high-resolution. In this figure, UV light activates the fluorophore and excitation is achieved with lasers at a specific wavelength. Only a small number of fluorophores are excited in each frame (see frames 1 and 2). After many cycles of stochastic activation and excitation, all fluorophores are bleached. (b) The Gaussian distribution of the PSF of an emitting fluorophore. The precise location of the fluorophore can be determined from the point of maximal intensity in the distribution (marked by a cross). (c) The precise location of the fluorophores in each frame can be determined with high precision and all frames combined to show the distribution of all fluorophores in the sample. From this, di/oligomers can be quantified by, for example, near-neighborhood analysis. Localizations are identified, which are within defined search radius “r” of a localized fluorophore in an iterative fashion. Once a localization has been assigned to a particular complex it is not included in further analysis. In the example here, receptors 1 and 2 (blue) are a dimer, receptors 3–6 are in a tetramer (green) and receptors 7–9 are monomers (orange).

The diffraction limit of light in conventional optical microscopy techniques restricts its ability to resolve structures below 200 nm. Several microscopy methods have overcome the diffraction limit, including single-molecule localization microscopy (SMLM), which individually localizes molecules, allowing the determination of the location of molecules with high precision as their point spread functions (PSF) do not overlap. In SMLM techniques, this is usually done by temporally separating emissions from individual fluorophore molecules to avoid PSF overlaps and allow resolutions of 20 nm or less (Fig. 2). Imaging of cells in TIRF mode allows visualization and activation of fluorophores only located at the plasma membrane, resulting in a higher signal-to-noise ratio. Following computational processing of all frames, individual-activated fluorophores are localized to determine their specific coordinates. When all the localizations from all frames are combined into a single image, appropriate filtering and downstream programs, such as near-neighborhood analysis, can be used to extract information from the precisely localized individual molecules (Lelek et al, 2021).

For the study of receptors, individual molecules must be labeled with fluorophores that are able to switch between excited “on” and “off” states. A range of different fluorophores can be used, including photoswitchable, photoactivatable, and spontaneously blinking dyes. There are a number of different strategies for labeling receptors with the fluorophores, including immunolabeling using conjugated antibody or nanobody dyes, encoding a tag or fluorescent fusion protein to your receptor of interest, or direct labeling approaches such as peptide labeling, or the use of unnatural amino acids to allow direct coupling of dyes by click chemistry (Betzig et al, 2006; Shcherbakova et al, 2014; Wang et al, 2014; Grimm et al, 2016; Neubert et al, 2018; Beliu et al, 2019; Han et al, 2021; Lelek et al, 2021). All types of SMLM are based upon the same principles, with the main difference between techniques being the fluorophore and the method used to achieve the blinking of individual molecules.

SMLM allows individual receptor molecules to be localized with high precision, which through appropriate postacquisition analysis, allows the oligomeric state and specific composition of oligomeric complexes to be determined. Additionally, it provides a map of the distribution of receptors across the cell membrane, which may reveal information about nanodomain formation or receptor clustering. Importantly, these techniques allow precise quantification of receptors at a range of receptor densities, allowing endogenous levels of the receptor to be assessed. However, SMLM approaches usually require fixed samples, long acquisition times, and careful analysis to avoid localization artifacts. These approaches allow insights into how receptor complexes are altered, either by forming different complexes or by changing the interfaces and interactions between receptors, in response to changes such as mutations, ligands, cell type, or receptor density.

**Fig. 3:**
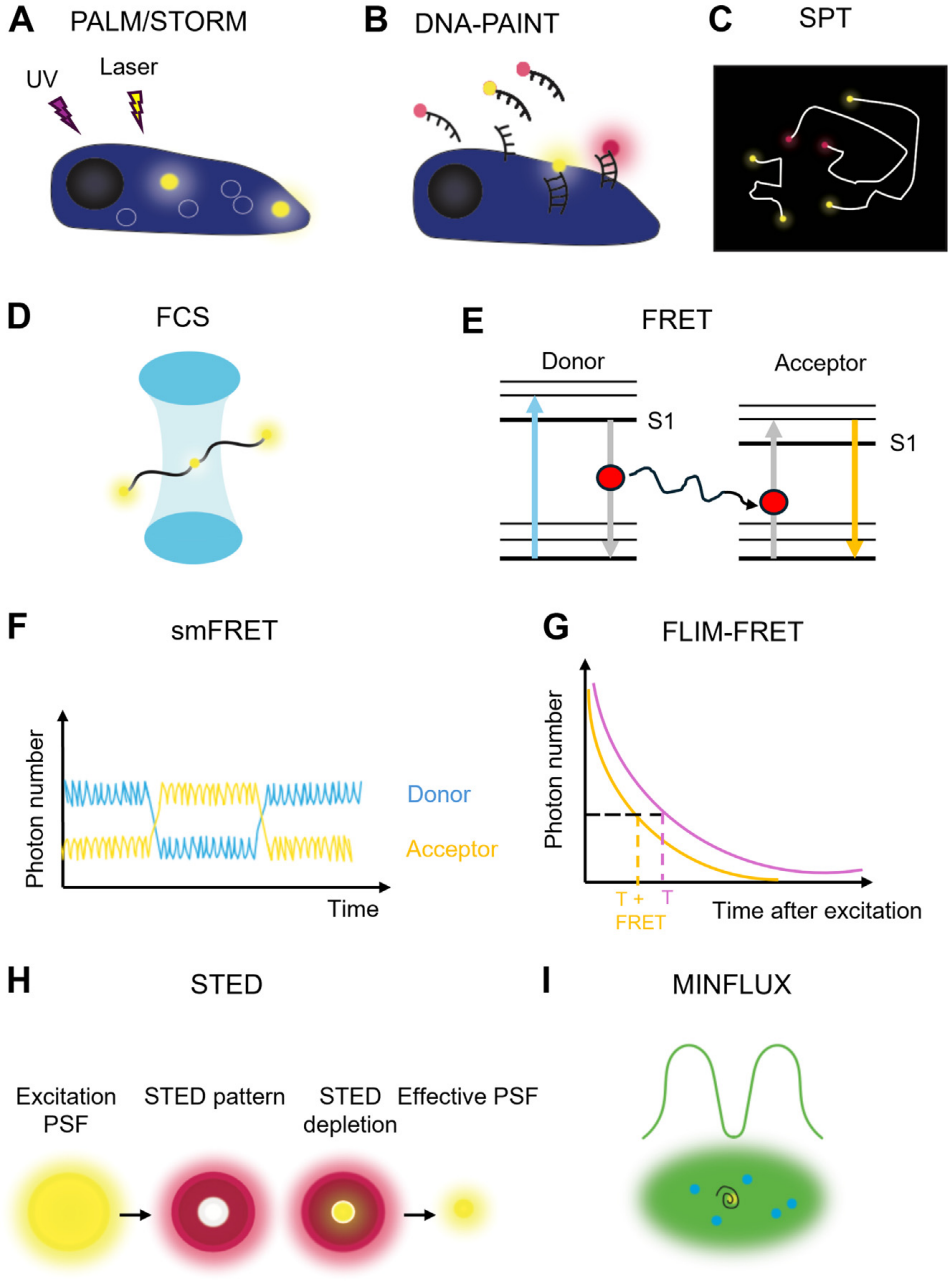
Commonly employed microscopy and spectroscopy approaches to study membrane protein receptor di/oligomerization. (a) Photoactivated localization microscopy (PALM) and stochastic optical reconstruction microscopy (STORM) use photoswitchable dyes or fluorophores to stochastically activate a small number of molecules at a time. Photoswitching is achieved in PALM by activation and excitation by UV light and lasers at a specific wavelength for the fluorophore respectively. Photoswitching in STORM is achieved by the exchange of reducing buffers to force fluorophores into the off state, causing blinking. (b) DNA point accumulation in nanoscale topography (PAINT) uses transient binding of complementary oligonucleotides, and reproduces blinking behavior by transitioning from diffusing to an immobilized state. (c) Single-particle tracking (SPT) involves tracking the diffusion of emitting fluorophores in a sparsely populated sample. (d) Fluorescence correlation spectroscopy (FCS) analyses the fluctuation and intensity of a fluorescent spot within a specific observation area (blue cylinder). (e) In fluorescence resonance energy transfer (FRET), a molecule is excited from the ground state when exposed to lasers at a specific wavelength (blue arrow), when molecules are in close proximity, resonance energy transfer occurs between donor and acceptor molecules (gray arrows) and fluorescence emission from the acceptor molecule upon returning to the ground state is measured (orange arrow). (f) Single-molecule FRET (smFRET) uses FRET to assess protein-protein interactions of individual molecules. The fluorescence of donor and acceptor molecules can be used to determine FRET efficiency between molecules. (g) Fluorescence lifetime imaging (FLIM) combined with FRET allows detection of fluorescent lifetimes of molecules without (T, purple) or with FRET (T+ FRET, orange), with FRET reducing the fluorescent lifetime of a molecule. (h) Stimulated emission depletion microscopy (STED) uses high laser power to deplete the fluorescence from most of the excitation region (red), to leave only a small area where the fluorophores are in the excited state (yellow), thereby reducing the effective point spread function (PSF) to below the diffraction limit. (i) MINimal photon FLUXes (MINFLUX) uses a donut-shaped excitation beam (green) to scan (represented by the black spiral) for the central minimum of an excitation region to find the precise location of the emitting molecule one fluorophore at a time. In the image, the blue circles are nonemitting molecules, whereas the yellow circle is the emitting molecule being localized.

The most commonly employed techniques to study membrane receptors are photoactivated localization microscopy (PALM), stochastic optical reconstruction microscopy (STORM), and point accumulation in nanoscale topography (PAINT; summarized in Fig. 3).

## 4 PALM and STORM

PALM was developed to use photoactivatable proteins, whereas STORM makes use of organic dyes that photoswitch in the presence of specific buffers (Betzig et al, 2006; Rust et al, 2006). PALM and STORM share the same principles of achieving high-resolution and PSF separation by stochastic excitation of a relatively small number of fluorophores in each frame imaged. PALM achieves this “blinking” through the activation and excitation of fluorophores using specific wavelengths of light, whereas STORM requires the exchange of buffers to reduce the organic dyes and cause blinking. The oligomeric state of the receptor can be determined using several different methods. For PALM, near-neighborhood analysis has been employed to determine receptors that are within a defined search radius distance (Fig. 2c). This enables the construction of a map of receptor monomers and homomers at the plasma membrane or, when 2-color imaging is carried out, a map of receptor heteromers (Jonas and Hanyaloglu, 2018). The use of transmembrane proteins that are known to only form monomers and/or dimers, such as CD86 and CD28, respectively, can provide additional controls for super-resolution imaging and analysis setups (Dorsch et al, 2009; Kasai et al, 2011).

These techniques have been used to study di/oligomerization of several different GPCR homomers and heteromers, including D2 dopamine receptor homomers, oxytocin, and prostaglandin E2 (OTR-EP2) heteromers and follicle-stimulating hormone receptor homomer and heteromers. This has been particularly useful in furthering understanding of how GPCR homomers and heteromers are influenced by ligand activation, including changes in dimer interface or the type of receptor complex formed (Jonas et al, 2015, 2018; Möller et al, 2020; Casarini et al, 2020; Agwuegbo et al, 2021; Walker et al, 2022; Sharrocks et al, 2024). PALM and STORM provide a level of detail on the specific receptor composition of complexes, such as dimeric or higher-order complexes, which had not been previously possible with other RET-based spectroscopy and microscopy approaches. PALM and STORM have also been used to monitor *β*_2_-adrenergic and Glucagon-like peptide-1 receptor clustering to nanodomains and metabotropic glutamate receptor organization at presynaptic active zones (Scarselli et al, 2012; Buenaventura et al, 2019; Siddig et al, 2020). STORM has also been used to monitor the GPCR dynamics through labeling ligands with fluorophores, rather than direct labeling of the receptor itself (Szalai et al, 2018). Labeling using fluorescent ligands has the advantage of having specificity to the receptor of interest and may facilitate the visualization of endogenous receptors in tissue samples, without the requirement for modifying the receptor.

Additionally, SPT has been combined with PALM to allow the monitoring of GPCR interactions with *β*-arrestins, which allows both high spatial and temporal resolutions (Manley et al, 2008; Eichel et al, 2018). In a recent study, a combination of SPT, dSTORM, and FLIM-FRET was used to investigate di/oligomerization of EGFRs and uncover the relative roles of different domains of the receptor in ligand-mediated dimerization (Mudumbi et al, 2024). The combination of approaches allows a high level of detail about receptor conformation to be explored. The role of higher-order ErbB oligomerization in receptor behavior and signaling as well as the impact of ligand type on ErbB receptor oligomerization and heterocomplex formation was investigated using STORM (van Lengerich et al, 2017). These approaches reveal the diversity of complex formation and can detect receptors at a range of densities, importantly allowing endogenous levels of receptors to be quantified. Additionally, even low abundance di/oligomers can be captured that may not be detected in other microscopy- or spectroscopy-based approaches.

### 4.1 PAINT

In contrast to PALM and STORM, PAINT uses dyes that freely diffuse and only appear as a PSF when immobilized at a target (Sharonov and Hochstrasser, 2006). The most common type of PAINT is DNA-PAINT, which uses fluorescently labeled DNA strands, which become immobilized when bound to the complementary target DNA strand. This transient event produces the “blinking” required for specific SMLM localization. DNA-PAINT can achieve resolutions similar to PALM and STORM and is particularly suited to multiple-color imaging. However, the stability of the DNA strand during imaging and nonspecific binding is a challenge, but advances in this area are beginning to mitigate these issues (van Wee et al, 2021). A quantitative form of DNA-PAINT has been used to quantify purinergic P2Y2 GPCR oligomers (Joseph et al, 2021). The binding kinetics of the imager and target strands allows highly accurate determination of receptor numbers correlating to single-molecule events observed, which prevents any overcounting that need to be carefully controlled and filtered out postacquisition in PALM and STORM approaches (Jungmann et al, 2016; Joseph et al, 2021).

Exchange-PAINT is another variation of this technique that allows multiplexing with multiple colors and is especially useful for studying receptor heteromer complexes. The approach uses a series of imager strands that are complementary to different docking strands on the sample, followed by washing steps and repeated imaging, which are eventually aligned and collated to form a final image with multiple colors (Jungmann et al, 2014). Using this approach in combination with SPT, MET-EGFR heteromers were demonstrated for the first time and the promotion of these complexes following ligand activation in cancer cell lines was monitored, giving both high-resolution static and dynamic information about these complexes (Harwardt et al, 2020). Additionally, PAINT has been used in combination with FRET to assess EGFR dimerization following ligand activation in live cells (Winckler et al, 2013).

## 5 Conclusion and future directions

Super-resolution microscopy, and in particular SMLM approaches, is an important tool to allow high-resolution detail of receptor di/oligomers to be uncovered, including determining the specific composition of complexes. By employing these techniques to study receptor di/oligomerization, detailed insights into specific oligomer composition and conformations in response to ligands, mutations, and cell environment have been gained.

Every technique has its advantages and limitations. One of the advantages of SMLM approaches such as PALM and STORM is the ability to map the cell surface landscape at varying densities, whereas FCS or SPT image smaller fields of view and/or under conditions where receptors are underexpressed or labeled to provide the single-molecule resolution. Another key advantage of SMLM is the ability to uncover the precise di/oligomer composition of receptor complexes. The most appropriate technique for a specific experimental question may depend on the cell context, sample thickness, receptor density, and requirement for receptor dynamics and/or live cell imaging to be explored. The combination of methods may take advantage of the best features of specific techniques to achieve high spatiotemporal resolution and uncover specific dynamics and conformations of oligomeric receptor complexes. Historically, the majority of dimerization studies have been conducted by heterologous expression of recombinant receptors (Fessl et al, 2023). The employment of new labeling approaches that allow endogenous receptors to be monitored, such as the use of gene editing to incorporate tags, will importantly allow the relevance of receptor di/oligomers in native cells to be explored. Additionally, emerging small-tagging approaches, including using site-specific labeling approaches such as the use of unnatural amino acids and click chemistry, avoid the need for bulky tags or antibodies that may interfere with receptor dimerization and will further the capabilities of the microscopy methods discussed here (Neubert et al, 2018; Han et al, 2021).

Emerging techniques such as MINimal photon FLUXes (MINFLUX), focused ion beam-scanning electron microscope, super-resolution cryocorrelative light and electron microscopy, and resolution enhancement by sequential imaging have the potential to achieve higher resolution than conventional SMLM techniques, even down to the Ångström scale. In particular, MINFLUX is a promising technique that combines some of the best features of SMLM approaches and stimulated emission depletion microscopy, another type of super-resolution microscopy, to allow 1–5 nm resolution to the size of the molecules themselves (summarized in Fig. 3, h and i; Balzarotti et al, 2017; Vicidomini et al, 2018; Reinhardt et al, 2023). This could allow receptor di/oligomer interactions to be resolved in even more detail and with ultrastructural detail of the surrounding membrane/cellular environment.

Although the organization of receptors at the plasma membrane has been the focus of previous work, quantifying receptor di/oligomers in intracellular compartments such as endosomes at the single-molecule level could be an interesting area of research. It is well established that both GPCRs and RTKs can signal from endosomes following endocytosis and can produce distinct signaling profiles from these intracellular compartments, compared with the plasma membrane (Wiley and Burke, 2001; Calebiro et al, 2009; Ferrandon et al, 2009; Sposini and Hanyaloglu, 2017; Stoeber et al, 2018; Klauer et al, 2024). With current super-resolution microscopy approaches that use TIRF, it is challenging to reliably discriminate between receptors at the cell surface and those localized in endosomes. However, by the employment of nanoscopic approaches such as MINFLUX, it may be possible to develop methodologies that would allow receptor di/oligomerization in endocytic compartments to be uncovered. Understanding receptor complex formation beyond the plasma membrane could open up new avenues for targeting specific signaling pathways therapeutically.

The further development of small and specific labeling approaches, including the integration of machine learning to automate image acquisition and analysis will further expand the capabilities of super-resolution microscopy for the study of membrane protein receptor di/oligomerization at a high-throughput level. Given the critical importance of receptor-receptor interactions physiologically and pathophysiologically, there is the potential that a single-molecule lens can be applied to drug discovery approaches.

## Conflict of interest

The authors declare no conflicts of interest.
